# A scoping review of routinely collected linked data in research on gambling harm

**DOI:** 10.1038/s41746-025-01713-z

**Published:** 2025-06-04

**Authors:** Pippa Boering, Matthew Jones, Kishan Patel, Daniel Leightley, Simon Dymond

**Affiliations:** 1 School of Psychology, Swansea University, Swansea, UK; 2 Gambling Harm UK, London, UK; 3 Department of Population Health Sciences, School of Life Course & Population Sciences, King’s College London, London, UK; 4 Department of Psychology, Reykjavík University, Reykjavík, Iceland

**Keywords:** Human behaviour, Psychology, Diseases, Health care, Medical research, Risk factors, Signs and symptoms

## Abstract

Gambling harm is a global public health challenge. Gambling is often recorded in settings using routinely collected data (RCD). Linking of existing RCD affords numerous opportunities for policy-led research on gambling harm and early intervention. To date, no previous review has examined research describing data linkage of RCD and gambling. Here, we searched for peer-reviewed articles using data linkage methodology with RCD and measures of gambling, gambling harm, or health-related outcomes. After screening 2373 articles, we conducted a narrative synthesis of the 17 included articles. Studies described data from 2,136,966 individuals, most originated from Nordic countries, adopted a range of experimental designs, tended to link individual-level data with risk factors for physical and mental health harms, and defined gambling in diverse ways. Study quality was mixed. There exist numerous opportunities for further data linkage studies with RCD to both inform public policy and understand population-wide changes in gambling.

## Introduction

Routinely collected data (RCD) are data originally gathered from administrative and clinical records, and which may subsequently form the basis of further research^1–3^. In healthcare and research settings, analysis of RCD may provide increased statistical power due to the large sample sizes often involved, which helps improve the external validity and generalizability of findings. This affords opportunities for descriptive or analytical epidemiology of health-related problems, identification of potential risk or protective factors, and evaluation of treatment effects over and above more restrictive sample designs, such as randomized control trials, that are both time- and cost-effective with a range of populations^4,5^. There are, however, inherent limitations with forms of RCD obtained in real-world settings that rely on observational recording and where the resulting data quality may be poor (due to coding errors, such as incorrect recording in notes, or an inaccurate assumption on the ordering of diagnoses). Yet, the increased availability of electronic health records, registries, and clinical databases permits a nuanced, evidence-led understanding of individual and population-wide healthcare journeys and opens exciting vistas for research that warrant further attention^6^.

**Fig. 1:**
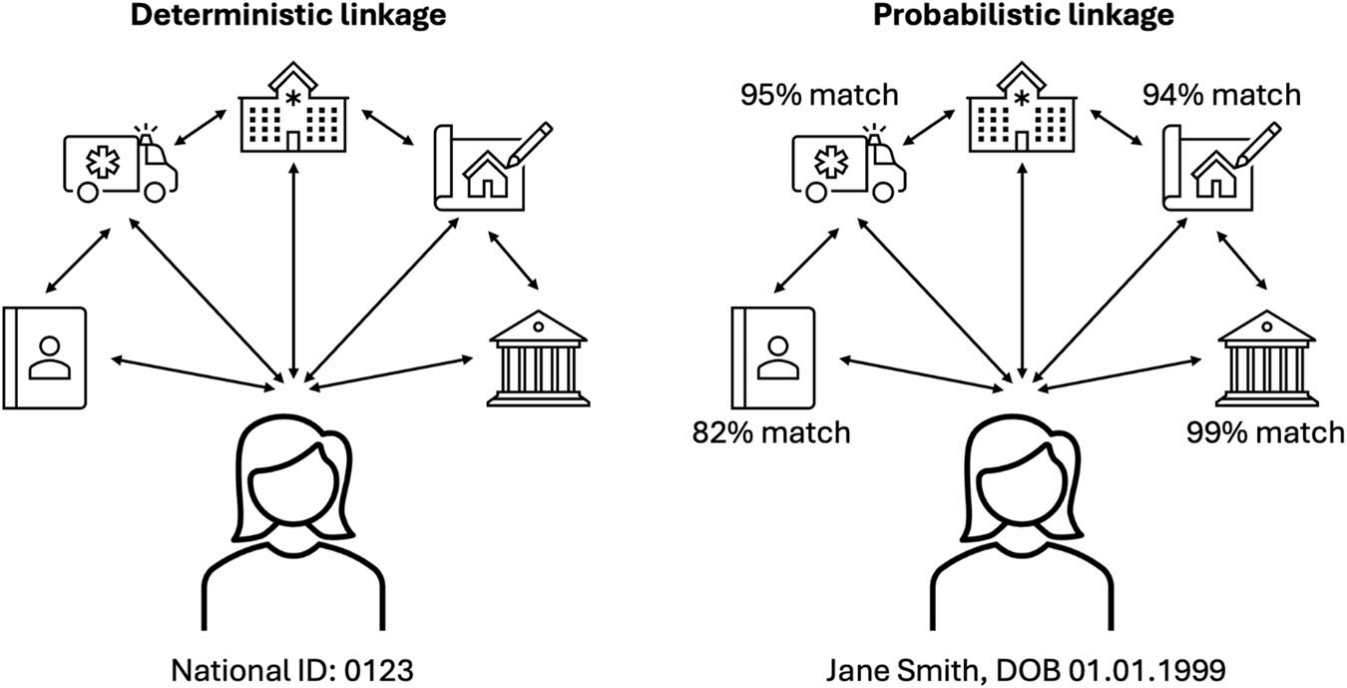
Graphical illustration of deterministic and probabilistic data linkage methods. The former methods use specifically defined rules to classify data sources, such as an individual’s identity number or date of birth, while the latter methods assign probabilistic weights to records conditional on a range of identifiers to represent the likelihood that they are drawn from the same individual.

Data linkage involves combining datasets of RCD to create a new data source^7,8^. Datasets that may be linked in studies with RCD include general patient population records, hospitalizations, national social insurance/welfare registries, employment records, prevalence surveys, occupational context, and death registrations. For instance, the secure anonymised information linkage (SAIL) databank holds anonymised data of the whole population of Wales, United Kingdom, from demographic, physical and mental health, mortality, and primary and secondary healthcare databases^9^. Three broad categories of methods are used to link data sources: deterministic (rule-based), probabilistic (score-based), and machine learning-based approaches^7,8,10^ (see Fig. 1). Deterministic methods use pre-existing specifically defined rules to classify data sources, such as an individual’s hospital identity number, date of birth, or postcode, while probabilistic methods assign weights to record pairs conditional on a range of identifiers to represent the likelihood that they are drawn from the same individual. It may not be possible to apply deterministic methods if an individual identifier is unavailable. In such instances, probabilistic methods provide a suitable, although less precise and more labor-intensive alternative, particularly for incomplete or error-prone data^10^. Machine-learning methods may be either supervised or unsupervised (based on training with prior datasets or not) and produce clusters of individuals at a higher degree of accuracy than other methods^11^. Regardless of method, in recent years, the use of data linkage in healthcare research has continued to increase as the availability and quality of data sources expand, supplemented by wider adoption of reporting guidelines like the reporting of studies conducted using observational routinely collected health data (RECORD) statement^1,12^.

Data linkage studies often use long-term data from multiple clinical encounters throughout the life course to provide valuable insight into health conditions and behaviors for research purposes^13^. Research using linked RCD not only illustrates the pragmatic nature of healthcare provision but enables researchers to efficiently access data recorded in real-time^5^. RCD linkage also facilitates the investigation of rarer conditions, such as suicidality^14^ and the use of a control group of people from the same source population who do not have the condition. Linkage methods have informed public health research, particularly regarding addictive behaviors and mental health conditions, such as evaluations of smoking cessation treatment^15^, opiate substitution treatment^16,17^, alcohol screening programs^18^, and the risk factors and pathways leading to suicidal behavior^19^. Although a growing body of evidence attests to the clinical and research utility of data linkage studies using RCD, there has been relatively minimal attention paid to their use in addiction research, particularly with behavioral addictions like gambling disorder (GD)^5,20^. A systematic review of this literature as it pertains to gambling harm and GD would therefore be timely.

Harm caused by gambling is recognized globally as a public health issue^21,22^. Gambling harms form part of the diagnostic criteria for GD within DSM-5 and ICD-11, in which both emphasize a persistent or recurrent pattern of gambling leading to adverse effects on health and wellbeing to individuals or to others, such as families, communities, and wider society. The prevalence of individuals who engage in problematic gambling or “who gamble in a manner that creates multiple problems that disrupt personal, family, financial, and employment circumstances”^21^ is estimated globally at 1.4% (95% CI: 1.06–1.84). A further 8.7% (95% CI: 6.6–11.3) of adults are estimated to be engaging in ‘any risk’’ gambling, which includes individuals “who meet the thresholds for problematic gambling or GD but also includes individuals who, at a minimum, report sometimes or occasionally experiencing at least one behavioral symptom or adverse personal, social, or health-related consequence from gambling”^23^.

Globally, the greatest risk profiles are evident among those engaged in online gambling^21^, and problematic patterns of gambling are associated with comorbidities, such as anxiety and depression, and increased risk of suicide^21–26^. For instance, estimates of suicidal ideation among people accessing gambling treatment vary between 22 to 81%^26^ and between 7 to 30% of individuals in clinical populations experiencing gambling harm report previous suicide attempts^27^. By way of comparison, among the general population, one study conducted in the UK found that up to 5% of people with experience of gambling harm report previous suicide attempts, compared to less than 1% of those without^22^. Many who die by suicide have had contact with primary and secondary healthcare services in the year before death^28–30^. Past-year contact with primary care settings can be as high as seven interactions^30^, while between two and five people are seen in secondary care settings like emergency departments—some as many as three times^29,31^. Contacts with primary and secondary healthcare form part of RCD and, as a result, there exist opportunities to exploit data linkage to better understand the benefits for research on gambling harm, comorbid conditions, and prevention/early-intervention programs.

There is increasing interest in the use of naturalistic, large datasets in research on gambling harm, such as operator data^32–34^, banking transaction data^35–37^, help-line data^38^, and geospatial data^39,40^. The development of new technology, such as new forms of online gambling and sports betting, and the widespread use of social media have all introduced novel sources of data for the analysis of gambling behavior^35,41–43^. This has afforded opportunities to investigate, for example, gambling operators’ use of social media^44–46^, population-wide trends in online searching for gambling^47^, natural language processing of online gambling treatment forums^48,49^, and fusing bank account transaction data via open banking with self-report gambling severity scores to identify risk profiles of these who did and did not experience gambling harm^50^. Clearly, the analysis of existing large datasets combined with advances in digital and financial technology is an innovative approach for policy-led gambling research and is capable of even wider dissemination with the inclusion of linked RCD.

To our knowledge, no prior work has sought to systematically synthesize the literature on the use of data linkage methods involving RCD in gambling research. Doing so confers considerable promise^20^ and insights into the status of the evidence base for policy-making research, as well as highlighting research gaps. Here, we sought to undertake the first scoping review of the use of routinely collected linked data in research on gambling harm. Our review included the following research questions:

1. What is the nature and extent of the literature investigating gambling harm using RCD?
2. How are datasets linked?
3. How are gambling harms defined?
4. What is the quality of the existing evidence?

## Results

### Study characteristics

**Fig. 2:**
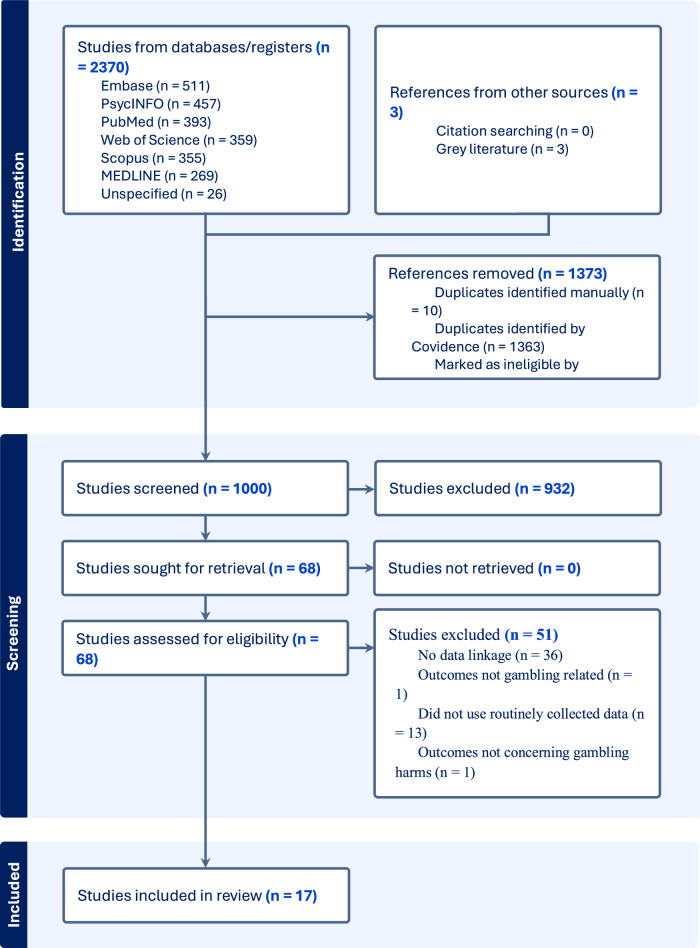
PRISMA flow chart of literature search and study selection process. Following identification of 2370 records, studies were screened against the inclusion criteria and resulted in 17 studies included in the present review.

The scoping review identified a total of 17 articles that met the inclusion criteria and were included in the final analysis. Figure 2 shows the PRISMA flow diagram and highlights that 14 articles were identified from literature searches and three further articles were included following expert consultation. The characteristics of the included studies are presented in Table 1.

**Table 1:** Characteristics of studies included in the scoping review

First author, year, (country)	Study design	Timeframe	Sample characteristics	Gambling screen	Sample size, age range, mean/median age, gender %	Data sources linked
**Aarestad et al.**^56^ **(Norway)**	Cross sectional	2008–2018	GD diagnosis = 5131, no GD diagnosis = 60,640	ICD-10 F63.0	*N* = 65,771, 18–88 years, mean age = 40.9, 18.2% female (*n* = 936).	Norwegian Patient Registry (NPR) and Social & Welfare Registry (FD-Trygd database)
**Bhatti et al.**^65^**(Canada)**	Cohort	2007–2014	Self-identified gambling = 16,002, no GD diagnosis = 14,650	PGSI ≥ 3	*N* = 30,652, 18+ years, 51.2% aged 35–65 (*n* = 15,872) 55% female (*n* = 16,897)	Canadian Community Health Survey (CCHS), Ontario Health Insurance Plan (OHIP), and the Canadian Institute for Health Information (CIHI) databases.
**Binde et al.**^53^ **(Sweden)**	Cohort	2015	Identified gambling = 2112, no GD diagnosis = 5127	PGSI ≥ 1	*N* = 7284, 18–67 years, mean age =43, 54% female (*n* = 3933)	Swedish Longitudinal Gambling Study (Swelogs) and Statistics Sweden registry data (SSYK)
**Fröberg et al.**^54^ **(Sweden)**	Cohort	2008–2010	2241 participants (3816 person-years)*	PGSI ≥ 3	*N* = 2241, 16–24 years, 43% of person-years* female (*n* = 1642)	Swedish Longitudinal Gambling Study (Swelogs) and Swedish National Agency for Education registry data
**Girard et al.**^53^ **(Norway)**	Case control	2008–2018	GD diagnosis = 5131, Diagnosis of psychiatric conditions = 30,476, Healthy control group = 30,164	ICD-10 F63.0	*N* = 65,771, 18–88 years, mean = 40.9, GD group comprised 18.2% women (*n* = 933).	NPR and the Statistics of Norway (SSB), Division of Welfare Statistics.
**Karlsson and Håkansson**^51^ **(Sweden)**	Cohort	2005–2016	GD diagnosis = 2099	ICD-10 F63.0	*N* = 2099, 18–83 years, mean age 36.5 years, 23% female (*n* = 474)	Swedish National Patient Register (SNPR), Swedish Cause of Death Register (CDR).
**Karlsson et al.**^52^ **(Sweden)**	Cohort	2011–2014	GD diagnosis = 848	ICD-10 F63.0	*N* = 848, 18–84 years, median age 38.23, 19.9% female (*n* = 169)	Swedish National Patient Register (NPR), Hospital Discharge Register, Swedish National Council for Crime Prevention, Register for SWPs, and Swedish CDR.
**Kaur et al.**^60^ **(Norway)**	Case control	2008–2018	GD diagnosis = 5131, no GD diagnosis = 22,289	ICD-10 F63.0	*N* = 27,420, mean age 41.2 years, 20.9% female (*n* = 4648)	NPR, Norwegian Prescription Registry (NorPD)
**Kristensen et al.**^25^ **(Norway)**	Cohort	2008–2021	GD diagnosis = 6899, no GD diagnosis = 391,897	ICD-10 F63.0	*N* = 398,796, mean age 36.8, 18.1% female (*n* = 1248)	Norwegian National Patient Registry (NPR), Norwegian CDR.
**Latvala et al.**^62^ **(Finland)**	Cross sectional	2015	At risk and problem gambling = 228** ;No GD: 603	PGSI ≥ 1	*N* = 831, 18–29 years, mean age 23.3, 48.5% women (*n* = 403)	Finnish Gambling 2015 Survey, Statistics Finland, Population Information Registry
**Latvala et al.**^61^ **(Finland)**	Cross sectional	2015	Weekly gambling = 137	PGSI ≥ 1	*N* = 676, 18–29 years, mean age 23.3, 43.4% female (*n* = 294)	Finnish Gambling 2015 Survey and Statistics Finland register data
**Latvala et al.**^63^ **(Finland)**	Cross sectional	2016–2017	Problem gambling = 139, at-risk gambling = 626, recreational gambling = 5003**, no gambling = 1310	PPGM ≥ 1	*N* = 7186, 18–75+ years, 33.1% 35–54 years (*n* = 2075), 52.3% female (*n* = 3910).	Finnish Gambling 2015 Survey, Statistics Finland social security register
**Laursen et al.**^67^ **(Denmark)**	Cohort	2000–2010	Problem gambling = 384, non-gamblers (non-problem and never gambled) = 18,241	2x positive answers on Lie/Bet questionnaire	*N* = 18,625, aged 20- > 50 + , 57.7% aged 50+ (*n* = 10, 196) 53.9% women (*n* = 10,037)	Danish Health and Morbidity Surveys, Danish National Criminal Register
**Reccord et al.**^64^ **(Canada)**	Cross sectional	1997–2016	History of gambling = 20, no history of gambling = 952	“History of gambling”	*N* = 972, aged 10–70+ years, mean age 41–44 years, 18.8% female (*n* = 223)	Newfoundland and Labrador Center for Health Information (NLCHI) suicide database, Vital Statistics annual mortality dataset and BLCHI client registry.
**Syvertsen et al.**^57^ **(Norway)**	Case control	2008–2018	GD = 5121, Illness control = 27,826, control = 26,695	ICD-10 F63.0	*N* = 59,642, 18+ years, mean age 31, 18.7% female (*n* = 11,166)	NPR and Social & Welfare Registry (FD-Trygd database)
**Syvertsen et al.**^58^**(Norway)**	Case control	2008–2018	GD = 5131, illness control = 30,476, control = 30,164	ICD-10 F63.0	*N* = 65,771, 18+ years, mean age 41, 18.5% female (*n* = 12,165)	NPR and Social & Welfare Registry (FD-Trygd database)
**Vestergaard et al.**^66^ **(Denmark)**	Case control	2013–2017	Diagnosed with GD = 1381, no GD = 1,381,000	ICD-10 F63.0	*N* = 1,382,381, 18+ median age 34, 12.9% female (*n* = 196,736)	Danish National Patient Registry, Danish Civil Registration System, Danish National Prescription Registry, Statistics Denmark, and Danish Health Service Registry

*Note*: *GD* gambling disorder, *DSM-5* Diagnostic and Statistical Manual of Mental Illnesses-5, *ICD-10* International Classification of Diseases 10th revision, *PGSI* Problem Gambling Severity Index, *Variables in this study were measured for the duration of time each individual contributed to the study, described as person-years. ** At risk (PGSI score 3–7) and problem gambling (PGSI score 8 + ).

### Study design and settings

Of the included studies, eight were based on cohort designs, five used cross-sectional designs, and four employed case-control designs. Most studies were conducted in Sweden (i.e., four)^51–54^, Norway (i.e., six)^55–59^^,^^60^, and Finland (i.e., three)^61–63^, with two studies each from Canada^64^^,^^65^ and Denmark^66^^,^^67^, and published between 2016 and 2025. Twelve studies focused on adverse outcomes associated with gambling, while five focused on risk factors associated with gambling behaviors.

### Sample characteristics

One study included participants aged 16 and above, with most including those aged 18 and older^54^. Ten studies consisted of mostly males, with the proportion of females ranging between 8.7% and 23%^25,51,52,55–58,64,66^. The remaining studies had an even distribution of gender^53,55,61–63,65,66^. Gambling harm was measured based on clinical diagnosis coded using ICD-10 in nine patient registry studies^25,51,52,55–58,67^ or the *Problem Gambling Severity Index* in five studies^54,61,62,65,68,69^. One study utilized self-reported gambling using the *Pathological Gambling Measure* (PPGM)^63^, and one used the *Lie/Bet questionnaire*^66^. A further study described an unspecified self-reported history of gambling^64^. The threshold for inferring harm from gambling using the PGSI varied between studies, with the majority including a score greater than one as “at risk and problem gambling”^53,61–63^, and one study including a score of greater than three as indicative of ‘problem gambling’^54^. Additional participant characteristics presented varied depending on the study, and included employment status, marital status, education level, and ethnicity (Table 1).

### Outcomes

A wide range of outcomes was included in this review. Most studies reported morbidity associated with gambling, including psychiatric diagnosis^52^, alcohol and smoking^51,52^, road traffic accidents (RTA)^65^, as well as physical comorbidity, such as chronic pulmonary disease^67^. Three studies reported on the association with suicide^51,59,64^. Further, eight studies described socio-cultural consequences of gambling, such as criminal activity^66^, changes in marital status^57^, unemployment and income^52,55,63^, and poor school achievement^54,61,62^. The remaining studies described risk factors for gambling harm, including ethnicity^56^, occupation^53^, unemployment^58^, and the role of gender^62^.

### Data linkage approaches

Most studies linked two datasets, with a minority of studies linking more than three and one containing five different datasets (Table 2). Deterministic linkage methodology was used in most studies, utilizing national identity numbers to link different records for one individual together^4,25,51,52,55–57,59,65,66,67,69,70^. One study linked data using a probabilistic method, based on the statistical similarity of data records^62^. No linkage method was described in five studies^54,58,61–63^. Four studies linked registry information to national surveys^54,65,66,69^. Studies based in Finland are all linked to the Finnish Gambling Survey and Statistics Finland^61–63^. Most studies (i.e., ten) linked to registers containing demographic information such as social insurance data^53,55,56,61–65,67^. Eight studies linked to patient registries^25,51,52,55–58,65^, four to mortality registries^51,52,59,64^, and two studies used crime registries^52,66^.

**Table 2:** Summary of data linkage methods

Study	Number of datasets	Linked to questionnaire/survey?	Linkage Method	Description of linkage methodology
**Aarestad et al.**^56^	2	No	Deterministic	Data from the two registries were linked using unique 11-digit National identity numbers.
**Bhatti et al.**^65^	3	Yes (CCHS)	Deterministic	Survey and administrative data were linked deterministically at the individual level using unique encoded identifiers and analysed at the Institute for Clinical Evaluative Sciences (ICES) in Toronto, Ontario.
**Binde et al.**^53^	2	Yes (Swelogs)	Deterministic	Swedish civic registration number based on date of birth and four extra digits.
**Fröberg et al.**^54^	2	Yes (Swelogs)	Unspecified	Register-based socio-demographic information was linked to the data (phone and postal interview/questionnaire).
**Girard et al.**^55^	2	No	Deterministic	Linked using participants national identity number.
**Karlsson & Håkansson**^51^	2	No	Deterministic	Linked using Swedish personal identification number.
**Karlsson et al.**^52^	4	No	Deterministic	Linked using personal identification number.
**Kaur et al.**^60^	2	No	Deterministic	Data from the two registries were linked using unique 11-digit National identity numbers.
**Kristensen et al.**^25^	2	No	Deterministic	Data from the two registries were linked using unique 11-digit National identity numbers.
**Latvala et al.**^62^	3	No	Unspecified	Registry data were linked with the Finnish Gambling 2015 data.
**Latvala et al.**^61^	2	No	Unspecified	NA
**Latvala et al.**^63^	2	No	Unspecified	The survey data were linked with the social security register data administered by Statistics Finland.
**Laursen et al.**^67^	2	Yes (Danish Health and Morbidity Surveys)	Deterministic	Linked using personal identification numbers.
**Reccord et al.**^64^	3	No	Probabilistic	Linkage between suicide data set and annual mortality dataset and client registry not described. Postal code to geographic area linkage is described for census data (demographics).
**Syvertsen et al.**^57^	2	No	Deterministic	Data from the two registries were linked using unique 11-digit National identity numbers.
**Syvertsen et al.**^58^	2	No	Unspecified	Data from the two registries were linked using unique 11-digit National identity numbers.
**Vestergaard et al.**^66^	5	No	Deterministic	Data were linked on an individual level across databases using the unique personal identification number.

### Definitions of gambling harm

A range of methods were used to define gambling harm and related constructs. Eight studies used the ICD-10 code F63.0 for ‘pathological gambling,’’ six studies used PGSI scores obtained from survey data, and one used scores on the Lie/Bet questionnaire or the PPGM, respectively.

### Quality assessment

**Fig. 3:**
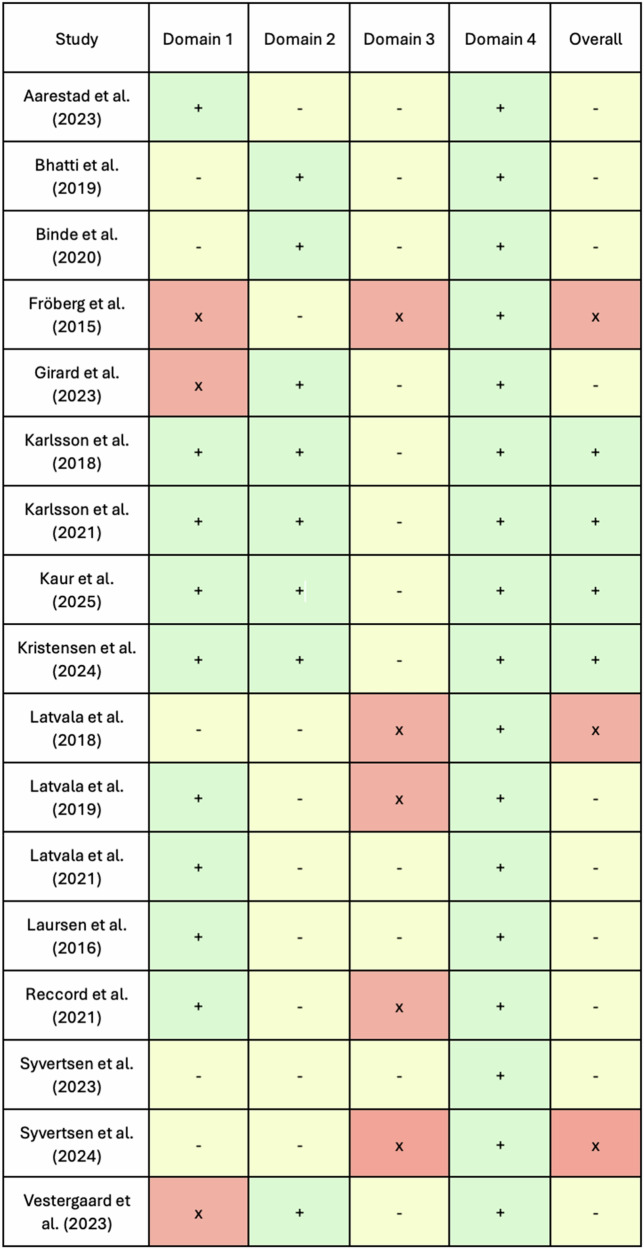
Graphical illustration of the quality assessment of included studies. The four assessed domains included description of the datasets linked, variables included in each study and sources of bias, the linkage process, and ethics approval. Note: + (green) denotes good quality, - (yellow) average quality, and x (orange) poor quality.

The results of the quality assessment are summarized in Fig. 3 and Supplementary Table 3. Of the included studies, four were good quality, ten were medium quality, and three were poor quality. Quality of each linkage study was assessed across four domains; 1) description of the datasets which were linked in each study, 2) variables included in each study and sources of bias, 3) the linkage process, and 4) ethics approval. All studies fulfilled domain 4 by gaining prior ethics approval. No studies achieved good quality in domain 3, with insufficient details of linkage methods and any changes to coding systems and potential sources of bias.

### Narrative details of the included studies

Aarestad et al.^56^ assessed the relationship between ethnicity and risk of gambling harm using NPR and the Norwegian social insurance database. Gambling harm was defined as a registered diagnosis of GD based on ICD-10. Second-generation individuals from minority ethnic groups, including Asian, African, and North American countries of birth, were at an increased risk of GD compared to the rest of the population of Norway^56^.

Bhatti et al.^65^ linked the CCHS, OHIP, and CIHI databases to determine the risk of RTAs among people who gamble. It was found that those at the highest risk (i.e., defined by PGSI scores greater than three) were at increased risk of RTAs compared to those who did not gamble^65^.

Binde et al.^53^ assessed gambling risk among different occupational groups using the Swedish Longitudinal Gambling Study (Swelogs 2015) and Statistics Sweden registry data. Gambling risk was defined as a PGSI score of three or more. Males working manual jobs were found to be at increased risk^53^.

Two studies reported findings related to educational attainment. Froberg et al.^54^ linked the Swedish Longitudinal Gambling Study survey (Swelogs) and Swedish National Agency for Education registry data and described an increased risk of gambling associated with poor school achievement among Swedish youth (16–24-year-olds)^54^. Latvala et al.^61^ linked results from the Finnish Gambling Survey with statistics Finland registry data to assess the association between gambling and school attainment among Finnish adults. Gambling risk was defined as a PGSI score of more than one. It was found that those with low grade point average (GPA) attainment scores were more likely to play daily lottery games and use online casinos compared to those with average and high GPA^61^.

Two studies reported findings related to employment and income. Girard et al.^55^ used linked registry data in Norway from the NPR and Statistics of Norway (SSB) to assess the relationship between income and gambling harm. Patients diagnosed with GD, as defined by ICD-10, were more likely to have lower annual income compared to the general population^55^. Latvala et al.^63^ investigated the role of social disadvantage with gambling severity using linked data from the Finnish Gambling Harms Survey and Statistics Finland social security registry. This study employed the PPGM as a gambling harm measure and demonstrated that harm was more common among people who were unemployed or received social security benefits^64^.

Three studies reported findings related to suicidality and gambling. Karlsson and Håkansson^51^ demonstrated an association between GD and increased mortality, suicidality, and comorbidity. This study linked the Swedish NPR and the Swedish CDR. Gambling harm was defined as a GD diagnosis as coded by ICD-10^51^. Reccord et al.^64^ demonstrated a significant association between completed suicide and gambling history. This study linked the NLCHI suicide database, the Vital Statistics annual mortality dataset, and the BLCHI client registry. Gambling was defined as having “a history of gambling” (p.920)^64^. Kristensen et al.^25^ used the NPR and CDR to assess suicide risk associated with GD, as well as 12 other patient groups, compared to the general population^59^. Suicide was the leading cause of death for people diagnosed with GD.

Kaur et al.^60^ described the relationship between the use of antidepressant medications and the likelihood of developing GD. The study compared participants diagnosed with GD and age- and gender-matched non-gambling individuals using linked data from the NPR with the NPR. It was found that the odds of being diagnosed with GD were almost three times greater among those individuals prescribed antidepressant medication^60^.

Latvala et al.^62^ demonstrated that gambling, defined by a PGSI score of more than one, was associated with smoking and risky alcohol use among men and with smoking among women. This study linked results from Finnish Gambling 2015, Statistics Finland, and the Population Information Registry^62^.

Laursen et al.^67^ linked Danish Health and Morbidity Surveys with the Danish National Criminal Register. Gambling was defined as two positive answers on the *Lie/Bet questionnaire*, and the authors found a significant association between problem gambling and increased criminal activity. No increase was detected in economic crime compared to other crimes^66^.

Syvertsen et al.^57^ linked the NPR with the Social & Welfare Registry (FD-Trygd) to demonstrate a reduced incidence of marriage and an increased risk of divorce among people with a GD diagnosis, as defined by ICD-10^57^. A subsequent paper by the same authors used the NPR and the Social and Welfare Registry (FD-Trygd) to assess unemployment as a risk factor for harmful/disordered gambling^58^.

Some studies reported outcomes related to multiple domains of inquiry, including clinical diagnoses, medication receipt, criminal behavior, and receipt of benefits. Karlsson et al.^52^ linked the Swedish NPR, Hospital Discharge Register, the Swedish National Council for Crime Prevention, the Register for Social Welfare Payments, and Swedish CDR. Analysis showed that a diagnosis of GD was associated with an increased prevalence of social welfare payments, criminal conviction, and diagnosis of psychiatric conditions, including intentional self-harm disorders^52^. Vestergaard et al.^66^ linked five registries: the Danish National Patient Registry, the Danish Civil Registration System, the Danish National Prescription Registry, Statistics Denmark, and the Danish Health Service Registry. The authors demonstrated an increased burden of mental and physical comorbidity among individuals with GD and increased use of prescribed medications and likelihood of criminal sentencing^67^.

## Discussion

The present scoping review identified 17 RCD linkage studies investigating gambling harm and a range of demographic and social-psychiatric factors. Gambling harm tended to be defined through either clinical diagnosis of GD or self-reported problem gambling severity, and a wide range of study designs were adopted. Most studies originated from Nordic countries and the overall quality was mixed. In total, we identified 27 linked datasets from primary and secondary healthcare settings and national/social insurance data, including social welfare information, population-wide prevalence surveys, and national mortality data. Deterministic linkage methods based on national identity numbers were most used to link two datasets, while some studies linked between three and five datasets. Analyzed timeframes ranged between one and 19 years and captured data from a combined population of 2,136,966 individuals across five countries.

This review demonstrates knowledge gaps in the literature on gambling harm in relation to linked RCD. We found RCD linkage studies with individuals who were predominantly identified as male, ranging in age between 16 and 88 years old. Across the included studies, men were more likely to be diagnosed with GD^53,60^ and experience adverse outcomes including RTA^65^ and low educational attainment^54^, while women were more likely to experience financial instability^55^ and concomitant psychiatric disorders^52^. Young people who engaged in gambling were at increased risk of unemployment, financial instability^54,55^ and early mortality^51^. Population-wide studies of gambling harm like these are critical for identifying demographic risk factors that may make some individuals more likely to experience harm than others. They also offer prevention and early intervention opportunities in settings routinely recording these demographic factors, such as further education and employment providers, financial advice and debt management services, and mental health screening and assessment agencies. The predictive relationships are evident, and the well-powered analyses on which they are based increased the likelihood of generalization to other jurisdictions.

Of the studies included in this review, only three assessed clinical comorbidities. Two of these studies investigated the association between gambling and suicidality^51,59,64^, while Vestergaard et al.^66^ found higher incidence of psychiatric comorbidity among people diagnosed with GD compared to those without a diagnosis^67^. Karlsson et al.^52^ demonstrated an increased prevalence of psychiatric conditions, including intentional self-harm disorders associated with people diagnosed with GD^52^. Kristensen et al.^25^ demonstrated that people with GD had an increased risk of suicide compared to the general population^59^. The relationship between harms experienced from gambling and psychiatric comorbidities is complex and potentially bidirectional, whereby the onset of specific disorders like depression or anxiety may either precede or follow problematic patterns of gambling^71^. It remains an important research challenge with the broader field of gambling studies to better elucidate the temporal relationships involved in problematic gambling and comorbidities^72^, and linked RCD studies may be uniquely placed to aid such investigations of assumed bidirectionality. For instance, longitudinal designs may permit an examination of the onset and time course of gambling harms and comorbid disorders^73^.

As outlined, data linkage methods are widely used in biomedical and public health research on a host of conditions relevant to gambling, such as suicide. For instance, Karlsson and Håkansson^51^ linked national registry data in Sweden with hospital admissions, medical appointments, and cause of death data to reveal a 15-fold increased risk of suicide in those with a diagnosis of GD. Of the 1024 patients admitted and the 5236 patient appointments reviewed, it was found that 55% of patients had a primary diagnosis of GD in either a primary or secondary care setting^51^. Unfortunately, the predictive role of these healthcare contacts was not further explored. No further studies were identified describing patient healthcare journeys, clinical trajectories, or analysis of contacts with healthcare settings. It is known that the link between past-year healthcare contact and suicide is both robust and a valuable means of informing suicide prevention^74^. Linkage of RCD may therefore provide a valuable data source for analysis of gambling-related suicidality, with large sample sizes, increased statistical power, and greater predictive utility when controlling for under-reporting and comorbidities.

Our review found a range of methods to define disordered or problematic gambling and gambling harm. Eight studies used the ICD-10 code, F63.0, for ‘pathological gambling’’ recorded in patient registries and typically had fewer participants defined as “gambling” compared to studies using survey data. Six studies defined gambling severity or harm using PGSI scores obtained from survey data, and one used the Lie/Bet questionnaire or the PPGM, respectively. The relative ubiquity of the PGSI as a proxy measure of gambling-related harm is hardly surprising; it is seen by the research community as the gold standard measure of gambling severity and associated negative consequences or harm. The PGSI was not, however, intended to be a measure of gambling-related harm^75^, although seven of its nine items do refer to the consequences (i.e., harms) experienced from gambling^76,77^. While specific measures of gambling harms have been developed^78^, none were employed by the data linkage studies reviewed here. As a result, people experiencing lower levels of gambling harm may therefore be under-represented in the included studies. Conversely, although the validity and accuracy of the PGSI is widely accepted^79^, variable inclusion thresholds observed between studies may limit the reliability or generalizability of results. Four studies defined problem gambling as a PGSI score of one or higher, and two defined this as having a score of three or higher.

This review included three studies describing socio-demographic characteristics associated with gambling, where gambling was defined using PGSI scores. The majority of PGSI questions relate to negative consequences of gambling and, operationally, we used it here to infer gambling harms as defined in the inclusion criteria^78^. We acknowledge that the use of the PGSI as a proxy for gambling harms is not widely accepted and that alternative scales exist that operationalize the health-harming impacts of gambling more explicitly^80,81^. While it is beyond the remit of the present scoping review to address the relative merits of the different gambling harm measures available, we support distinguishing between the language used to define gambling harm, hazardous gambling, GD, and problem (or problematic) gambling. The predominance of the PGSI in the studies included in this review demonstrates that much work remains to be done in challenging this orthodoxy. We not only encourage future data linkage research on gambling to adopt a person-centered approach aimed at reducing the stigma surrounding gambling^23^ but also to consider a range of diagnostic outcome measures that capture the continuum of gambling and related harms.

We undertook a quality assessment of the current state of linked RCD research on gambling harm. The included studies were assessed as demonstrating a range of quality, with no studies obtaining maximum scores (Fig. 3). All described the datasets linked, including their purpose and type, as well as the original data collection method. However, few studies described the percentage of the population from which the data were derived or any quality assurance process to ensure high-quality, representative data. Although most studies described a deterministic linkage process using national identification numbers to link data sets, the full details of the linkage process, such as specific changes to coding systems and data quality assessment, were often inadequately described. Indeed, it is noteworthy that one of the included studies identified following expert consultation was previously excluded for not explicitly describing the linkage method. Methods exist to evaluate linkage quality and inform the likelihood of rates of missed links, false links, and any clustering or errors with relevant variables of interest^82^. Future data linkage research with RCD should consider describing the outcome of any linkage quality techniques applied to the data and account for potential variability in subsequent analysis. Moreover, since none of the present studies referred to RECORD guidelines^1,12^ for the reporting of data linkage, we encourage both data linkers and data analysts to consider the wider adoption of reporting standards and practices in their work. Doing so will not instill confidence in data linkage methods as powerful research tools but should foster wider dissemination and increased uptake.

Data linkage studies in gambling research should make explicit that and how linkage was conducted—indeed, we have highlighted this as a research and knowledge gap and contend that there is an opportunity for the development of reporting guidelines specifically for studies conducted on gambling. We note that all studies received ethical approval and included formal declarations of interest, where relevant. Overall, to promote wider adoption of data linkage methods in gambling research and enhance reliability and generalizability of findings, future studies should describe more fully the linkage methods involved, incorporate machine learning-based analysis of large gambling datasets^83,84^, justify the assumed representativeness of the population(s) studied, and highlight all data management and quality assurance procedures followed. Our review noted an absence of machine learning methods in the linkage of datasets, which perhaps may not be surprising given concerns in the context of patient privacy, the use of personal identifiers, and how they may be used^85^. However, machine learning does present unique opportunities to reduce the risk of bias in the linkage of data parameters, but may also lead to a high false positive rate. One solution may be to consider using machine learning as a verifier of linkages obtained via deterministic and probabilistic methods. Further research should evaluate this possibility.

Our findings indicate that the use of conflicts of interest statements, funding declarations, and adoption of open science practices in data linkage RCD gambling research was limited. Nine studies reported conflicts of interest (Table 3), all but one of the 17 studies described a funding source, and none were funded by the gambling industry. The role of industry funding in gambling harms research and any subsequent impact on public health policy should, at a minimum, necessitate that any conflicts of interest are disclosed, and that industry funding should be avoided^23^.

**Table 3:** Conflicts of interest and funding source reported in studies

Study	Conflict of interest reported?	Nature of conflict of interest	Funding source
**Aarestad et al.**^56^	Yes	Author received research funding from Norsk Tipping (a gambling operator owned by the Norwegian government) and GambleAware, a charitable body, which funds its research program based on donations from the gambling industry. The author also undertakes consultancy for various gambling companies in the area of player protection and social responsibility in gambling.	This study was funded by the Research Council of Norway (grant number 273718). Open access funding provided by University of Bergen.
**Bhatti et al.**^65^	No		This work was supported by Sunnybrook Research Institute intramural funds. Investigators were supported by the Canadian Institutes of Health Research, Canada Research Chair in Medical Decision Sciences (Redelmeier).
**Binde et al.**^53^	No		Project funded by the Public Health Agency of Sweden, financed by the Swedish Research Council for Health, Working Life and Welfare (Forte).
**Fröberg et al.**^54^	`Yes	The author has received personal fees from the Swedish organization of online gambling companies (BOS), personal fees from Svenska Spel (Swedish state-owned gambling company), and Play among friends, an NGO-owned Finnish gambling company, outside the submitted work.	The Public Health Agency of Sweden funded the Swedish Longitudinal Gambling Study.
**Girard et al.**^55^	Yes	An author has received research funding from Norsk Tipping and GambleAware. The author undertakes consultancy for various gambling companies in player protection and social responsibility in gambling.	The present study was funded by the Research Council of Norway, grant no. 273718. The authors have no other financial relationships relevant to this article to disclose.
**Karlsson & Håkansson**^51^	No		No financial support was received specifically for this study. An author holds a position as professor at Lund University financed in collaboration between Lund University and the Swedish gambling operator monopoly, Svenska spel AB.
**Karlsson et al.**^52^	Yes	An author holds a position as professor at Lund University financed in collaboration between Lund University and the Swedish gambling operator monopoly, Svenska spel AB. Another author has received a grant from the same gambling operator monopoly, Svenska Spel AB as part of Svenska Spel ABs’s responsibility for gambling research.	This research was funded by the Swedish Southern Health Care Region Research (grant 2020-0424, GD—associations with suicidality, economic vulnerability, and mortality) and from AB Svenska Spel, the Swedish state-owned gambling operator (grant FO 2019-0013 GD—associations with psychosocial problems, suicide, and crime).
**Kaur et al.**^60^	Yes	An author has received research funding from Norsk Tipping and GambleAware. The author undertakes consultancy for various gambling companies in player protection and social responsibility in gambling	Funded by the Research Council of Norway (No. 273718).
**Kristensen et al.**^25^	Yes	An author has received research funding from Norsk Tipping and GambleAware. The author undertakes consultancy for various gambling companies in player protection and social responsibility in gambling.	Study was funded by the Norwegian Competence Center for Gambling and Gaming Research and the faculty of Psychology at the University of Bergen.
**Latvala et al.**^62^	No		The Ministry of Social Affairs and Health, Finland, and the Finnish Foundation for Alcohol Studies funded the study (appropriation under section 52 of the Lotteries Act).
**Latvala et al.**^61^	No		The Ministry of Social Affairs and Health, Finland, and the Finnish Foundation for Alcohol Studies funded the study (appropriation under section 52 of the Lotteries Act).
**Latvala et al.**^63^	No		The Gambling Harms survey was funded by the Ministry of Social Affairs and Health, Finland, within the objectives of the 52 Appropriation of the Lotteries Act.
**Laursen et al.**^67^	No		This work was funded by the Danish Agency for Science, Technology and Innovation.
**Reccord et al.**^64^	No		This study was funded by a grant from the Newfoundland and Labrador Support Unit for People and Patient-Oriented Research and Trials (NL SUPPORT).
**Syvertsen et al.**^57^	Yes	An author has received research funding from Norsk Tipping and GambleAware. The author undertakes consultancy for various gambling companies.	Open access funding provided by University of Bergen. The study was funded by the Research Council of Norway (no. 273718).
**Syvertsen et al.**^58^	Yes	An author has received research funding from Norsk Tipping and GambleAware. The author undertakes consultancy for various gambling companies.	The study was funded by the Research Council of Norway (no. 273718).
**Vestergaard et al.**^66^	Yes	In 2020–2022, the clinic provided several gambling operators with an expert appraisal in online gambling patterns The clinic was paid for these advisory services by the standard tariff at Aarhus University Hospital.	Open access funding provided by Aarhus University Hospital. This work was supported by a grant from the Ministry of the Interior and Health of Denmark.

We failed, however, to identify any open science practices in the included studies, which may reflect the heterogeneity of study designs, the relative novelty of the field of data-linkage gambling research, and potential data-access restrictions. While it was not the intent of the present review to gauge the adoption of open science practices here, clearly, the field of gambling research has much work to do. The implementation of open science methods aims to improve research quality and reduce publication bias^86,87^ and further linked RCD studies are encouraged to consider adoption of open science practices wherever feasible.

The review did not apply any geographical restrictions with its search criteria and found that the extant data linkage gambling research with RCD was overwhelmingly conducted by Nordic countries^88,89^. In contrast to the relative paucity of research from other countries, the burgeoning literature using linked RCD from Nordic countries like Sweden, Denmark, Norway, and Finland may reflect differences in clinical coding practices or the impact of different gambling landscapes, such as the public gambling monopolies or private licensing systems operating within these countries and their impact on research. In Europe, only Finland and Norway currently operate fully public monopoly models of gambling, and there is a relative paucity of research evaluating their effectiveness at reducing gambling harm. The available evidence suggests that monopolies may have lower estimated prevalence rates of problematic gambling and overall reduced levels of gambling participation (total consumption) compared to private licensed regimes^90^. Other countries identified in our review, like Denmark and Canada operate state-owned or state-controlled monopolistic companies which tend to operate increasingly in a commercial or expansionist manner^69,91^. It is noteworthy, therefore, and perhaps unsurprising that most of the studies included in this review originated in Nordic countries like Sweden, Norway, and Finland with state-owned operators and access to large datasets of RCD for research and prevention purposes.

Linkage models vary by country and research environment, and this may affect the risk of linkage error, selection bias, and data completeness as well as the ability to conduct linkage studies^4,10^. Individuals in Nordic countries access tax-funded and public health care systems similar to the UK, and which remain valuable sources of research data, as our findings confirm^88^. The personal identification numbers used in Nordic countries to access healthcare services enable the deterministic linkage of multiple RCD sources, reducing the risk of misclassification and incomplete linkage^92^. By contrast, countries, such as the UK, have seen repeated proposals to link healthcare records from primary and secondary care since 2012, yet progress has been hindered by concerns over data transparency, governance challenges, and public trust^6,93,94^. This notwithstanding, the reviewed studies primarily include male participants from Nordic countries, which poses limitations in terms of the wider relevance and generalizability of the findings, particularly among more ethnically diverse or economically varied populations. It is of paramount importance that future data linkage studies on gambling ensure as representative a sample as possible and for cross-cultural comparisons to be undertaken where data recording systems allow.

Since the Covid-19 pandemic, access to linked data has improved, notably for health surveillance. However, our findings underscore that the adoption of linked RCD in gambling research remains limited, and further investigation is needed to understand how different linkage methodologies impact data reliability and generalizability^93^. Selection bias may also influence gambling research outcomes, as individuals with gambling-related harms may not always engage with healthcare services, leading to underrepresentation in linked datasets. Moreover, the lack of systematically collected gambling-related data in UK healthcare settings presents a significant limitation, constraining opportunities to develop robust evidence and targeted interventions^91^. The lack of evidence from the UK may imply that researchers in the UK face additional barriers to working with RCD and that gambling-related data are not widely collected in UK healthcare settings, which clearly limits research opportunities.

Linkage of RCD may provide novel datasets to assess the socio-economic costs of gambling harm and determine intervention cost-effectiveness^95,96^. However, it is recognized that linked data alone does not establish causation. While some studies included in the review did assess the economic cost of gambling among occupational groups, the impact of gambling on income, and role of economic hardship as a risk factor for self-harm among people with a diagnosis of GD^52,53,55^, the social and economic costs were not calculated. That is, the studies reviewed did not link to datasets of aggregated or patient-level costs and healthcare utilization activities^52,53,55^. Vestergaard et al.^66^ did, however, assess the health costs of GD and mental and somatic comorbid conditions and found that gambling was associated with an estimated attributable cost of illness and welfare services of €4.0 and €17.6 M of indirect attributable costs due to reduced productivity calculated using the human capital approach^67^. It is possible to adopt this approach and undertake secondary analysis of costs by using estimates of, for instance, the social costs of certain occupational groups^53^ or social welfare and criminal justice costs^52^. Estimating the costs of gambling harm in future linked RCD studies either directly or indirectly via secondary analysis may generate novel insights and policy-led research opportunities involving financial (banking) transactions, affordability, and gambling behaviors^95^. These financial insights may have valuable implications for policy.

There are policy implications of using data linkage research to improve public health interventions for gambling and comorbid conditions. Linked healthcare record data could, for instance, help develop early intervention programs by identifying individuals at risk of gambling harm. These data could also support monitoring systems that track gambling-related risk factors over time, enabling more proactive harm reduction approaches. Enhanced collaboration between gambling regulators, healthcare providers, and financial institutions could improve intervention effectiveness by combining financial data, self-exclusion registries, and healthcare records. Overall, the burgeoning work on data linkage we identified here alongside similar developments in data fusion and Big Data analytic techniques involving financial transactions and industry provided customer dataset may have enormous potential for policy-led research^20,50^; we call for wider research consensus on how these innovative data-led synergies may be optimized to tackle gambling harm.

A final research gap concerns the finding that only one study investigated the role of ethnicity and GD, highlighting barriers to accessing care, which may prevent or delay diagnosis^56^. Future data linkage research with RCD should consider strategies to promote inclusion and diversity in public health research on gambling. For instance, a better understanding is needed of ethnicity and gambling harm among minority communities and individuals from deprived backgrounds^97,98^. Tracking patient healthcare utilization journeys in large datasets of RCD will help to inform prevention and early intervention opportunities.

The present review may have limitations. A risk of bias analysis, while not standard practice in scoping reviews, was not conducted. Our critical appraisal assessment may, therefore, have been limited by the absence of quality thresholds; we were, however, able to qualitatively appraise included studies to demonstrate methodological robustness. Our unrestricted systematic search was followed by a snowballing method of identifying included studies and may have omitted relevant papers. Similarly, we did not include the gambling gray literature in our search^99^.

The present scoping review is the first to describe research using linkage of RCD to investigate gambling harm. Most research was conducted in Nordic countries with unique gambling landscapes. Much of the evidence was focused on sociocultural factors, such as financial impacts, crime, and marriage, but few studies included in this review described health consequences associated with gambling. A growing number of data linkage studies examine the relationship between gambling suicide. Overall, our findings support the need to conduct research using linked RCD to further explore relationships between gambling harm, demographic, and mental health factors.

## Methods

We conducted a scoping review in accordance with Joanna Briggs Institute^99,100^ and the preferred reporting items for systematic reviews and meta-analysis extension for scoping reviews (PRISMA-ScR) guidelines^101^. The review protocol was pre-registered on Open Science Framework (DOI: 10.17605/OSF.IO/MEV68) and the completed PRISMA-ScR is included in Supplementary Table 2.

### Search strategy

The search strategy was developed by PB and MJ in consultation with an expert-by-experience, KP. Systematic searches with no specified timeframe were conducted of Medline, PubMed, Web of Science, Scopus, Embase, and PsycInfo databases using the search terms, ‘linkage,’’ ‘routine data,’’ ‘gambling,’’ and ‘specific gambling harm’’ (see Supplementary Table 1 for the full search terms). Articles identified from the search were uploaded to Covidence for further extraction. The reference lists of included studies were manually checked for additional studies that may fulfill the inclusion criteria.

### Inclusion and exclusion criteria

We screened articles using a full list of inclusion and exclusion criteria organized according to PICO (population, intervention/issue, comparison, and outcome) categories presented in Table 4. To be included, articles had to be in English and involve individuals experiencing gambling harm, problematic gambling, or GD, with any intervention, exposure, or comparator, measuring any gambling-related harm outcomes, and across time and/or settings. Articles had to involve data linkage of at least two independent databases. Studies using aggregated data were excluded due to the lack of linkage methodology, despite using registry data^73,102^.

**Table 4:** Inclusion and exclusion criteria

PICO	Include	Exclude
**Population**	Persons at risk of, or experiencing, gambling harms, including problematic gambling and GD.	No exposure to gambling in sample.
**Intervention/Exposure**	Any intervention or exposure.	-
**Comparators**	Any comparators.	-
**Outcomes**	Any gambling related harms or related health outcomes (e.g., gambling behavior, financial wellbeing, or mental health).	Outcomes unrelated to gambling
**Timings**	Any timings (e.g., pre- and post-intervention or exposure or cross-sectional).	-
**Settings**	Any clinical or supportive care settings (e.g., primary care or third sector addiction services).	-

*Note*: To be included, studies also had to describe a data linkage method with at least two databases of RCD.

### Article selection and data extraction process

Titles and abstracts were reviewed independently by PB and MJ. Full text manuscripts of selected citations were then obtained and assessed against eligibility criteria. Disagreements were resolved through discussion, and inter-rater reliability was high (89.8% agreement, kappa *p* = 0.171). Identified articles were shared and discussed with experts with experience of conducting data linkage research on gambling to ensure the findings were representative and up to date. One further, unpublished study was identified from this process. Data extracted included study design and methodology, data linkage methods, participant demographics, and outcome measures, including number of events and measures of association.

### Quality assessment

Study quality was assessed using accepted guidelines^103,104^. These guidelines assess four major domains, including 1) details regarding the datasets which were linked, 2) researcher-selected variables and sources of bias, 3) the linkage process, and 4) ethics approval. The first domain assesses each data set included in the linkage study independently, whereas the remaining domains summarize the study. Two authors (M.J., P.B.) assessed each study individually, and disagreement was resolved through discussion. Studies achieved points for each domain and were subsequently classified as ‘good,’’ ‘average,’’ and ‘poor’’ quality (see Fig. 3 for a graphical visualization of the quality assessment findings and Supplementary Table 3 for the ratings of individual studies).

## Supplementary information

Supplementary Information

## Data Availability

Data supporting this study are openly available from OSF at http://osf.io/mev68/ (DOI: 10.17605/OSF.IO/MEV68).

## Code availability

Code sharing is not applicable to this article as no code was generated during the current study.
