# Supplementary material for: A scoping review of routinely collected linked data in research on gambling harm
Source: NPJ Digit Med. 2025 Jun 4;8:331. doi: 10.1038/s41746-025-01713-z (PMC12137888; doi:10.1038/s41746-025-01713-z)
Supplement: Supplementary file 1 — Supplementary Information [file 41746_2025_1713_MOESM1_ESM.pdf]

Supplementary Information

Supplementary Table 1: Search terms.

S1	(linked OR linked-data OR data-linkage OR linkage)
S2	(data OR routine* OR “routine data” OR "routinely captured" OR "routinely collected" OR RCD OR “routinely acquired” OR “health data” OR “health-data” OR “hospital data” OR “GP data” OR “general practice data” OR “big data” OR “structured data” OR “unstructured data” OR “semi-structured data” OR “anonym* data” OR “mortality data” OR “death data” OR “coroner report data” OR “electronic health record*” OR “electronic patient record*” OR EHR OR EPR OR “digital patient record*” OR “digital health record” OR chart OR “chart review” OR record* OR clinical OR decedent)
S3	(“gambling” OR “gambler”)
S4	(harm* OR suicide* OR suicidality OR para-suicide OR self-injur* OR self-harm OR sequelae OR adverse OR depress* OR anxiety OR distress OR problem* OR consequence* OR impairment OR health OR wellbeing OR disorder* OR *cide)

Supplementary Table 2: PRISMA- ScR Checklist.

Section	Item	PRISMA-ScR checklist item	Reporting location
**TITLE**			
**Title**	1	Identify the report as a scoping review.	First page of the article
**ABSTRACT**			
**Structured summary**	2	Provide a structured summary that includes (as applicable): background, objectives, eligibility criteria, sources of evidence, charting methods, results, and conclusions that relate to the review questions and objectives.	Abstract
**INTRODUCTION**			
**Rationale**	3	Describe the rationale for the review in the context of what is already known. Explain why the review questions/objectives lend themselves to a scoping review approach.	Paragraph five of the Introduction
**Objectives**	4	Provide an explicit statement of the questions and objectives being addressed with reference to their key elements (e.g., population or participants, concepts, and context) or other relevant key elements used to conceptualize the review questions and/or objectives	Paragraph six of the Introduction
**METHODS**			
**Protocol and registration**	5	Indicate whether a review protocol exists; state if and where it can be accessed (e.g., a Web address); and if available, provide registration information, including the registration number.	Methods paragraph one
**Eligibility criteria**	6	Specify characteristics of the sources of evidence used as eligibility criteria (e.g.,	Table 4

		years considered, language, and publication status), and provide a rationale.	
**Information sources**	7	Describe all information sources in the search (e.g., databases with dates of coverage and contact with authors to identify additional sources), as well as the date the most recent search was executed.	Methods paragraph two
**Search**	8	Present the full electronic search strategy for at least 1 database, including any limits used, such that it could be repeated.	Methods paragraph four
**Selection of sources of evidence**	9	State the process for selecting sources of evidence (i.e., screening and eligibility) included in the scoping review.	Methods paragraph four
**Data charting process**	10	Describe the methods of charting data from the included sources of evidence (e.g., calibrated forms or forms that have been tested by the team before their use, and whether data charting was done independently or in duplicate) and any processes for obtaining and confirming data from investigators.	Methods paragraph four
**Data items**	11	List and define all variables for which data were sought and any assumptions and simplifications made.	Methods paragraph four
**Critical appraisal of individual sources of evidence**	12	If done, provide a rationale for conducting a critical appraisal of included sources of evidence; describe the methods used and how this information was used in any data synthesis (if appropriate).	Methods paragraph five + supplementary information
**Synthesis of results**	13	Describe the methods of handling and summarizing the data that were charted.	Methods paragraph five
**RESULTS**			

**Selection of sources of evidence**	14	Give numbers of sources of evidence screened, assessed for eligibility, and included in the review, with reasons for exclusions at each stage, ideally using a flow diagram.	Figure 2
**Characteristics of sources of evidence**	15	For each source of evidence, present characteristics for which data were charted and provide the citations.	Table 1, 2 and 3
**Critical appraisal within sources of evidence**	16	If done, present data on critical appraisal of included sources of evidence (see item 12).	Figure 3
**Results of individual sources of evidence**	17	For each included source of evidence, present the relevant data that were charted that relate to the review questions and objectives	Results section
**Synthesis of results**	18	Summarize and/or present the charting results as they relate to the review questions and objectives.	Results section
**DISCUSSION**			
**Summary of evidence**	19	Summarize the main results (including an overview of concepts, themes, and types of evidence available), link to the review questions and objectives, and consider the relevance to key groups.	Discussion paragraph one
**Limitations**	20	Discuss the limitations of the scoping review process	Discussion paragraph 11
**Conclusions**	21	Provide a general interpretation of the results with respect to the review questions and objectives, as well as potential implications and/or next steps.	Discussion paragraph 12
**FUNDING**			
**Funding**	22	Describe sources of funding for the included sources of evidence, as well as	Acknowledgements

		sources of funding for the scoping review. Describe the role of the funders of the scoping review.	

Supplementary Table 3: Quality assessment. *Note*: Good Quality = More than 75% requirements met; Average Quality = 50-75% of requirements met; Poor Quality = Less than 50% of requirements met.

Criteria			Study										
			Aaresaad 2023	Bhatti 2019	Binde 2020	Fröberg 2015	Girard 2023	Karlsson 2018	Karlsson 2021	Kaur 2024	Kristensen 2024	Latvala 2018	Latvala 2019
Domain 1	Purpose of dataset given?	Dataset 1	1	1	1	1	1	1	1	1	1	1	1
		Dataset 2	1	1	1	1	1	1	1	1	1	1	1
		Dataset 3		1					1			1	
		Dataset 4							1				
		Dataset 5											
	Specified dataset type?	Dataset 1	1	1	1	1	1	1	1	1	1	1	1
		Dataset 2	1	1	1	1	1	1	1	1	1	1	1
		Dataset 3		1					1			1	
		Dataset 4							1				
		Dataset 5											
		Dataset 1	0	1	1	1	1	1	1	0	1	1	

	Stated coding system?	Dataset 2	0	0	1	0	0	1	1	1	1	1	1
		Dataset 3		0					1			1	
		Dataset 4							1				
		Dataset 5											
	Stated % population coverage?	Dataset 1	1	1	0	0	0	1	1	1	0	0	0
		Dataset 2	1	0	0	0	1	0	1	1	1	0	0
		Dataset 3		0					0			0	
		Dataset 4							1				
		Dataset 5											
	Described data collection methods?	Dataset 1	1	1	1	1	1	1	1	1	1	1	1
		Dataset 2	1	1	0	0	0	1	1	1	1	0	1
		Dataset 3		1					1			1	
		Dataset 4							1				
		Dataset 5											
	Described quality	Dataset 1	0	0	0	0	0	0	0	0	0	0	0

	assurance process?	Dataset 2	0	0	0	0	0	0	0	0	0	0	1
		Dataset 3		0					0			0	
		Dataset 4							0				
		Dataset 5											
Domain 2	Specified participant inclusion criteria?		0	1	1	1	1	1	1	1	1	1	1
	Specified variables used for linkage?		1	1	1	0	1	1	1	1	1	0	0
	Specified changes to coding system?		0	0	1	1	0	0	0	0	0	0	0
	Specified sources of bias?		1	1	1		1	1	1	1	1	0	1
Domain 3	Specified intended linkage precision?		0	0	0	0	0	0	0	0	0	0	0
	Description of linkage method given?		1	1	1	0	1	1	1	1	1	0	0
	Measure of quality of		0	0	0	0	0	0	0	0	0	0	0

	linked data given?											
Domain 4	Did the study receive ethical approval?	1	1	1	1	1	1	1	1	1	1	1

Supplementary Table S3: continued.

Criteria			Study					
			Latvala 2021	Laursen 2016	Reccord 2021	Syvertsen 2023	Syvertsen 2024	Vestergaard 2023
Domain 1	Purpose of dataset given?	Dataset 1	1	1	1	1	1	1
		Dataset 2	1	1	1	1	1	1
		Dataset 3						1
		Dataset 4						1
		Dataset 5						1
	Specified dataset type?	Dataset 1	1	1	1	1	1	1
		Dataset 2	1	1	1	1	1	1
		Dataset 3						1
		Dataset 4						1
		Dataset 5						1
	Stated coding system?	Dataset 1	1	1	1	1	1	1
		Dataset 2	1	1	1	1	1	0
		Dataset 3						1
		Dataset 4						0
		Dataset 5						0
	Stated % population coverage?	Dataset 1	1	0	1	0	0	0
		Dataset 2	0	1	0	0	0	0
		Dataset 3						0
		Dataset 4						0
		Dataset 5						0

	Described data collection methods?	Dataset 1	1	1	1	0	1	1
		Dataset 2	0	1	1	1	1	0
		Dataset 3						1
		Dataset 4						0
		Dataset 5						0
	Described quality assurance process?	Dataset 1	1	0	0	0	0	0
		Dataset 2	0	0	0	0	0	0
		Dataset 3						0
		Dataset 4						0
		Dataset 5						0
Domain 2	Specified participant inclusion criteria?			1	1	1	1	1
	Specified variables used for linkage?			0	0	0	0	0
	Specified changes to coding system?			1	1	0	1	0
	Specified sources of bias?			1	0	1	0	0
Domain 3	Specified intended linkage precision?			0	0	0	0	0
	Description of linkage method given?			1	1	0	1	0
	Measure of quality of linked data given?			0	0	0	0	0
Domain 4	Did the study receive ethical approval?			1	1	1	1	1
